# Medial patellofemoral ligament reconstruction using an autograft or allograft for patellar dislocation: a systematic review

**DOI:** 10.1186/s43019-019-0008-0

**Published:** 2019-08-23

**Authors:** Kyung Wook Nha, Ji Hoon Bae, Sun Chul Hwang, Young Jun Nam, Myung Jin Shin, Nikhl N. Bhandare, Aseem Kumar, Dong Geun Kang, Dong Yeong Lee

**Affiliations:** 1 Department of Orthopaedic Surgery, Inje University Ilsan Paik Hospital, Ilsan, Republic of Korea; 2 Department of Orthopaedic Surgery, Korea University, Guro Hospital, Seoul, Republic of Korea; 3 Department of Orthopaedic Surgery, Gyeongsang National University Hospital, Jinju, Republic of Korea; 4 Department of Orthopaedic Surgery, Bhandare Hospital, Panaji, India; 5 Department of Orthopaedic Surgery, St. Stephen’s Hospital, Delhi, India; 6 Department of Orthopaedic Surgery, Gyeongsang National University Changwon Hospital, Changwon, Republic of Korea; 7 Department of Orthopaedic Surgery, The Armed Forces Daegu Hospital, Daegyeong-ro 425-41, Hayang-eup, Gyeongsan-si, Gyeongsangbuk-do 38427 Republic of Korea

**Keywords:** Knee, Patellar instability, Medial patellofemoral ligament, Autograft, Allograft, Systematic review

## Abstract

**Purposes:**

The purpose of this study is to review the use of an allograft or autograft in medial patellofemoral ligament (MPFL) reconstruction.

**Materials and methods:**

Various electronic databases were searched for relevant articles published from January 2000 to September 2017 that evaluated clinical outcomes of MPFL reconstruction using an autograft or allograft. Data search, extraction, analysis, and quality assessments were performed based on Cochrane Collaboration guidelines.

**Results:**

The study of 21 autografts and one allograft was included in this review. Although direct comparative studies were unavailable, the Kujala score and subjective results were reported in the majority of these studies. While the use of an autograft for MPFL reconstruction yielded satisfactory clinical outcomes with few perioperative complications, no new outcome has been drawn from the use of allografts.

**Conclusions:**

Although many studies have shown favorable clinical results for MPFL reconstruction using an autograft, the clinical results of MPFL reconstruction using an allograft have not yet been sufficient to achieve meaningful clinical results due to low levels of evidence. Direct comparisons were not conducted because there were very few studies on allografts; thus, further research in this area should be performed in the future.

**Electronic supplementary material:**

The online version of this article (10.1186/s43019-019-0008-0) contains supplementary material, which is available to authorized users.

## Introduction

Recurrent patellar dislocation has an annual incidence rate ranging from 5.8 to 77.8 per 100,000, with the highest incidence rate being in young and active people [1–3]. Failure to treat patellar dislocation can lead to patellar instability, persistent knee pain, and patellofemoral osteoarthritis eventually. Hence, appropriate treatment is needed.

Regarding patellar dislocation, the medial patellofemoral ligament (MPFL) plays a critical function in the patellofemoral joint as a primary stabilizer. Treating a patellar dislocation is challenging for orthopedic surgeons due to the complex procedures required and possible unsatisfactory results such as frequent recurrence. Although medial soft-tissue realignment surgery is the conventional treatment to medialize the patella, these procedures do not reconstruct or repair the MPFL. A rather high recurrent instability rate of 27% has been reported after medial capsule reefing [4–7].

Recent studies have indicated that MPFL reconstruction is associated with favorable clinical outcomes [8–11]. Bitar et al. [12] reported that treatment with MPFL reconstruction using a graft produced good results, based on the analyses of postoperative recurrences and the better final clinical score results. Despite previous results, when surgeons perform MPFL reconstruction using a graft, there is debate regarding graft choice, particularly on whether an autograft or an allograft should be used.

Previous studies have reported clinical outcomes of MPFL reconstruction using an autograft such as a semitendinosus, a patellar tendon, or a gracilis tendon [12–16]. Mikashima et al. [17] have suggested that autografts are better than allografts because they can achieve better results using an autogenous tendon without anything surpassing it in terms of autologous histocompatibility. Conversely, Hohn et al. [18] suggested that the use of an allograft can preserve autogenous tissue and may be preferable in patients with connective tissue disorder or ligamentous laxity. They found that MPFL reconstruction using allograft tissue resulted in a low risk of recurrent instability, perhaps comparable to what has been published by others who have used autograft tissue. In the same vein, some authors have reported that allograft tissues have some advantages over autografts in terms of donor-site morbidity, including loss of strength, faster recovery, decreased surgical time, and use in patients with connective tissue disorder [19–21]. Despite several graft-fixation methods having been used for different types of graft, no consensus has been reached about the ideal kind of graft.

To clarify these discrepancies and establish evidence for selecting graft materials for MPFL reconstruction, the purpose of this study is to review the use of an allograft or autograft in MPFL reconstruction. We hypothesized that both autograft and allograft materials would yield favorable results for MPFL reconstruction.

## Materials and methods

### Literature search

We used multiple comprehensive databases to find studies that reported clinical outcomes of MPFL reconstruction using an autograft or an allograft for patellar dislocation. This study adhered to the Cochrane Review Methods. Reporting was conducted in accordance with Preferred Reporting Items for Systematic Reviews and Meta-Analyses (PRISMA) Statement. To identify relevant studies, controlled vocabulary and free-text words described in Additional file 1 were used to search MEDLINE, EMBASE, the Cochrane Central Register of Controlled Trials, Web of Science, and SCOPUS databases between January 2000 and September 2017. Due to the recent development of surgical techniques and equipment, past research results that are too old may have a heterogeneous effect on recent research results. Thus, only studies after the year 2000 were included and analyzed. All relevant studies were identified regardless of language, publication type (article, poster, conference article, instructional course lectures, etc.), publication journal, or publication year. This search was updated in September 2017, including reference lists of studies and any review articles identified. Reference lists of the investigated studies were scrutinized to identify any possible additional publications not found through electronic or manual searches. In cases of two or more studies by the same author, we determined whether patients had been “duplicated.” If duplicated, only the latest study was included.

### Eligibility criteria

Studies were included in our investigation according to the following eligibility criteria: (1) subjects were humans who had received MPFL reconstruction using an autograft or an allograft, (2) studies that evaluated clinical outcomes of MPFL reconstruction, and (3) researchers conducted level-I, -II, -III, or -IV evidence studies. Studies were excluded if they did not evaluate the effect of surgical technique, focused on revision surgery, included patellar dislocation after total knee arthroplasty, had subjects with congenital disease, or connective tissue disorders, only reported non-clinical outcome measures or intra-operative measures, consisted of level-V evidence (case report, technical note, and letters to editor), were review articles, animal studies, or in vitro studies. Detailed criteria are summarized in Table 1.

**Table 1:** Inclusion and exclusion criteria

**Inclusion criteria**	
The subjects that received MPFL reconstruction using autograft or allograft were human	
The studies evaluated the clinical outcomes of MPFL reconstruction	
Studies reporting a minimum 2-year follow-up data on clinical outcomes	
Level-I, -II, -III, or -IV evidence required	
No exclusions were made on the basis of language	
Studies on this topic which were published since the year 2000	
**Exclusion criteria**	
Studies that did not evaluate the clinical outcomes of MPFL reconstruction	
Studies regarding revision surgery	
Patellar dislocation after total knee arthroplasty	
Subjects who had congenital disease or connective tissue disorders	
Combined surgery for treatment other ligament injury such as ACL, PCL, collateral ligament injuries	
Studies reporting less than 2-year follow-up data on clinical outcomes	
Level-V evidence (case report, technical note, letters to editor), review articles	
Animal studies or in vitro studies	

*MPFL* medial patellofemoral ligament. *ACL* anterior cruciate ligament, *PCL* posterior cruciate ligament

### Data collection and analysis

Two authors independently assessed the titles or abstracts of studies identified with the search strategy. Subsequently, a full paper review was conducted for the final inclusion. Uncertainty regarding the study inclusion was resolved through discussion and consensus. Data were extracted by authors using predefined forms. They were then checked for accuracy. We extracted data of study characteristics and patient demographics (Table 2). Clinical outcomes, such as the Kujala score (mean and standard deviation (SD) of preoperative and postoperative score), Lysholm score, Tegner score, redislocation rates (at final follow-up), instability episodes, subjective results, reoperation rates, range of motion (ROM), and perioperative complications, are revealed in Table 3.

**Table 2:** Characteristics of the included studies on medial patellofemoral ligament reconstruction for patellar dislocation using an autograft versus an allograft

Study	Journal	Study design	Level of evidence	Year	Sample size (knees)	Age (years)	Sex (M: F)	Graft type	Patellar fixation	Femoral fixation	Follow-up Time (months)
Astur et al. [15]	Open Orthop J	RCS	3	2015	Autograft: 58	29.8 (18–45)	30: 28	Gracilis (58)	Transpatellar tunnel with Endobutton	Interference screw	Minimum 24
Bitar et al. [12]	Am J Spots Med	RCT	1	2011, 2012	Autograft: 21	24.0 ± 6.3	12: 9	Patellar tendon (21)	Suture	Interference screw	44
Deie et al. [22]	Knee Surg Sports Traumatol Arthrosc	Case series	4	2005	Autograft: 46	19.2 (6–43)	9: 34	Semitendinosus (46)	Transpatellar tunnel	MCL pulley	114 (60–144)
Deie et al. [13]	Am J Spots Med	Case series	4	2011	Autograft: 31	22.2 (12–34)	5: 24	Semitendinosus (31)	Suture	Femoral socket pullout suture	38 (24–60)
Dragoo et al. [23]	Am J Spots Med	PCS	2	2017	Allograft: 8	36.3 ± 8.7	1: 7	Semitendinosus (8)	Suture anchor	Interference screw	51 (25–79)
Drez et al. [9]	Arthroscopy	Case series	4	2001	Autograft: 14	22 (14–52)	10: 5	ITB (3), Semitendinosus (6), Semitendinosus + gracilis (5)	Suture anchor	Suture to periosteum	31.5 (24–43)
Ellera Gomes et al. [11]	Arthroscopy	Case series	4	2004	Autograft: 16	26.7 (21–37)	4: 11	Semitendinosus (16)	Transpatellar tunnel	Osteoperiosteal tunnel	Minimum 60
Han et al. [16]	Arch Orthop Trauma Surg	Case series	4	2011	Autograft: 59	24.3 (15–41)	19: 33	Semitendinosus (51) Gracilis (8)	Transpatellar tunnel	Interference screw	68.4 (37–85)
Kang et al. [24]	Am J Spots Med	RCT	2	2013	Autograft: 82	28.9 ± 5.2	32: 50	Semitendinosus (82)	Suture	Interference screw	24
Kang et al. [14]	Knee Surg Sports Traumatol Arthrosc	Case series	4	2014	Autograft: 45	26.6 ± 5.8	18: 27	Semitendinosus (45)	Soft-tissue tunnel	Interference screw	33.7 ± 8.4
Ma et al. [25]	Arthroscopy	RCT	2	2013	Autograft: 32	28.4 ± 4.2	10: 22	Semitendinosus (32)	Suture anchor + suture	Interference screw	40 (24–55)
Mikashima et al. [17]	Acta Orthop Belg	Case series	4	2006	Autograft: 24	21.8 ± 4.9	10: 14	Semitendinosus (24)	Anchor or transpatellar tunnel	Femoral tunnel with Endobutton	41.0 ± 8.7
Niu et al. [26]	Int J Clin Exp Med	RCT	2	2016	Autograft: 22	27.5 ± 4.8	10: 12	Semitendinosus + gracilis (22)	Suture anchor	Interference screw	48
Nomura et al. [27]	Arthroscopy	Case series	4	2006	Autograft: 12	24.8 ± 10.6	4: 8	Semitendinosus (12)	Transpatellar tunnel	Screw and spiked washer	50 ± 10
Panni et al. [28]	Am J Spots Med	Case series	4	2011	Autograft: 48	28 (16–60)	11: 37	Semitendinosus (48)	Transpatellar tunnel	Interference screw	33 (24–54)
Ronga et al. [29]	Am J Spots Med	Case series	4	2009	Autograft: 28	32.5 ± 11.4	21: 7	Semitendinosus (5) Gracilis (23)	Transpatellar tunnel	Interference screw	37 (30–48)
Schottle et al. [30]	Knee Surg Sports Traumatol Arthrosc	Case series	4	2005	Autograft: 15	30.1 (19–36)	4: 8	Semitendinosus (15)	Suture anchor	Interference screw	47.5 (24–70)
Torisuka et al. [31]	Knee	Case series	4	2011	Autograft: 20	23 ± 8	9: 11	Semitendinosus (15)	Transpatellar tunnel	Femoral tunnel with endobutton	30 (24–53)
Vavalle et al. [32]	J Orthop Traumatol	Case series	4	2016	Autograft: 16	22 (18–25)	9: 7	Quadriceps (16)	Rectus femoris insertion	Anchor	38 (28–48)
Wang et al. [33]	Int Orthop	RCS	3	2013	Autograft: 70	25 ± 8	27: 43	Semitendinosus + gracilis (70)	Suture anchor	Interference screw	48
Witonski et al. [34]	Biomed Res Int	Case series	4	2013	Autograft: 10	27.2 ± 8.1	4: 6	Patellar tendon (10)	Suture	Anchor	43 (24–55)
Zhao et al. [35]	Am J Spots Med	RCT	2	2012	Autograft: 45	25.0 ± 6.6	8: 37	Semitendinosus (45)	Transpatellar tunnel	Interference screw	60

*RCS* retrospective cohort study, *RCT* randomized controlled trial, *PCS* prospective comparative study, *ITB* iliotibial band, *MCL* medial collateral ligament

**Table 3:** Clinical outcomes of the included studies on medial patellofemoral ligament reconstruction for patellar dislocation using autograft versus allograft

Study	Group (*n*)	Clinical outcomes	Complications
Astur et al. [15]	Autograft: 58 Endobutton (30) Anchor (28)	There were no statistical differences among postoperative Kujala, Fulkerson, and SF-36 questionnaire scores between the endobutton and anchor fixation groups. In the endobutton group, there were favorable outcomes to shorter follow-up length (2–5 years) compared to those with a longer follow-up length (5–10 years) for both Kujala and Fulkerson scores, but no difference for the anchor fixation group	No recurrent dislocation or subluxation 1 patellar fracture in the endobutton group 3 patients had subjective complaints of discomfort in the endobutton group due to endobutton prominence 2 patients developed postoperative arthrofibrosis, one for each technique
Bitar et al. [12]	Autograft: 21	Treatment with MPFL reconstruction using the patellar tendon produced better outcomes compared to non-operative treatment. The Kujala score was significantly higher in the MPFL reconstruction group, when compared with the mean value of the non-operative group. The MPFL reconstruction group presented a higher percentage of good/excellent results (71.4%) when compared with the non-operative group (25.0%)	No patellar recurrent dislocation or subluxation
Deie et al. [22]	Autograft: 46	There were significant differences between the preoperative and postoperative Kujala scores. Based on their results, they recommended MPFL reconstruction with the advancement of the vastus medialis or with Insall’s procedure. ROM was investigated as knee extension 0° ± 5° and knee flexion of 147° ± 3°	No recurrent patellar dislocation 4 knees had experienced the subluxation sensation and the apprehension signs remained
Deie et al. [13]	Autograft: 31	The Kujala score improved from 64 (range, 35–70) to 94.5 (range, 79–100). ROM improved for all patients, with knee extension 0° ± 2° and knee flexion of 145° ± 3°	No patellar redislocation 1 patient remained with a positive apprehension sign
Dragoo et al. [23]	Allograft: 8	Based on the KOOS, Lysholm, Tegner, and VR-12 scores, there were no statistically significant differences between the MPFL repair and MPFL reconstruction groups	No recurrent patellar dislocation There were no other surgical complications, such as stiffness, infections, painful hardware, or wound problems, at final follow-up
Drez et al. [9]	Autograft: 14	About 80% of patients showed excellent or good results and 14% of patients had fair or poor results. Based on Fulkerson’s functional knee score, 93% had excellent or good results. Postoperative mean Kujala score was 88.6 (57–100), Tegner activity level averaged 6.8 pre-injury and 6.7 postoperatively	10 patients had patellofemoral crepitus. 1 patient had medial facet tenderness 4 patients lost some flexion motion 9 patients had atrophy No apprehension sign
Ellera Gomes et al. [11]	Autograft: 16	According to Crosby-Insall criteria, about 94% of patients had excellent or good results. According to Aglietti criteria, about 88% of patients had excellent or good results	No infection and vascular problems were found 1 knee, the apprehension sign was positive, patellofemoral pain was present, and patellar tracking was abnormal Patellar crepitus was detected in 10 knees
Han et al. [16]	Autograft: 59	The average ROM was improved from 30 ± 2° to125 ± 5°. Both the mean Kujala score (41.4 versus 82.6) and the mean modified Cincinnati score (50.6 versus 88.7) were improved at recent follow-up. There were significant differences between preoperative and postoperative scores in both scales. In addition, the results of the very 2 scales were not associated with the presence of cartilage lesion, and sex	No patellar dislocation or subluxation No apprehension sign 3 knees developed postoperative stiffness, but resolved after 6 months of physical therapy
Kang et al. [24]	Autograft: 82 Y-graft (40) C-graft (42)	Y-graft group versus C-graft group: mean Lysholm score were 92.3 ± 3.9 and 88.4 ± 6.8 (significant). : Mean Kujala score 95.9 ± 4.7 and 91.3 ± 9.7 (significant). : Good or excellent rate of 97.5% in the Y-graft group compared with 83.3% in the C-graft group (significant) Thus, Y-graft technique had favorable outcomes compared to C-graft procedure	No recurrent dislocation or subluxation
Kang et al. [14]	Autograft: 45	The mean Lysholm score increased from 51.8 ± 6.2 to 91.7 ± 4.1 and mean Kujala score was from 53.4 ± 5.3 to 90.9 ± 6.6. There were significant differences between preoperative and postoperative scores	No recurrent dislocation or subluxation None remained with a positive apprehension sign after surgery
Ma et al. [25]	Autograft: 32	When MPFL reconstruction technique was compared to medial retinaculum plasty, medial retinaculum plasty yielded similar results to MPFL reconstruction for recurrent patellar instability. Median Kujala score improved from 54 (46–63) to 87 (78–100)' Median Tegner score improved from 3 (1–5) to 5 (2–8). There were no significant differences in Kujala, Tegner, and subjective questionnaire scores between medial retinaculum plasty and MPFL reconstruction groups. About 88% of patients had excellent or good results	In 3 patients (9%) in the MPFL reconstruction group, patellar lateral shift was observed that exceeded 1.5 cm but was less than 2.0 cm. 2 knees had mild anterior knee pain and limitation during flexion activities. A flexion deficit of less than 5° remained at final evaluation No extension deficit
Mikashima et al. [17]	Autograft: 24 Anchor (12) Patellar tunnel (12)	Postoperative mean Kujala score was improved from 30.5 ± 6.7 to 95.2 ± 12.9 (range, 82–100). About 76.5% of patients resumed sports activity at the previous level. Extensor and flexor strength of the affected knee to the unaffected knee were improved. The author recommended suturing to fibrous tissue and the patellar periosteum as the first choice	2 cases of patellar fracture 1 case had a persistent patellar apprehension sign
Niu et al. [26]	Autograft: 22	Mean Kujala score improved significantly from 56.7 ± 17.7 to 86.8 ± 14.4 at 48 months follow-up. Mean Lysholm score improved significantly from 59.9 ± 3.8 to 92.4 ± 1.9 at 48 months follow-up. The clinical outcomes of the MPFL reconstruction group are better than that of the medial retinaculum plasty group	No superficial wound infection No deep vein thrombosis and ROM limitation No patellar redislocation
Nomura et al. [27]	Autograft: 12	Kujala score improved from 61.7 ± 4.9 to 96.0 ± 5.2. According to the grading system of Insall, 83% of patients had excellent or good results and 17% of patients had fair results. There was no poor result	No recurrent dislocation or subluxation No positive apprehension sign No perioperative complications
Panni et al. [28]	Autograft: 48	Mean Kujala score improved significantly from 56.7 ± 17.7 to 86.8 ± 14.4. Mean Larsen score improved significantly from 12.4 ± 3.2 to 17.1 ± 2.7. Mean Fulkerson’s knee score improved significantly from 59.2 ± 21.8 to 90.1 ± 14.0. Mean modified Lysholm score improved significantly from 57.6 ± 19.6 to 88.1 ± 16.2. 87% of patients were either satisfied or very satisfied with the pain relief achieved	No patellar dislocation postoperatively
Ronga et al. [29]	Autograft: 28	Mean modified Cincinnati score increased from 52 ± 19 (range, 44–67) to 89 ± 21 (range, 74–100). Mean Kujala score increased from 45 ± 17 (range, 39–53) to 83 ± 14 (range, 74–91). Both clinical scales did not show significant differences in patients with and without osteochondral lesions. There were no significant differences in the Insall-Salvati Index between preoperative and postoperative results. The muscle volume of the thigh of the operated limb increased with time, but remained less well developed than those of the non-operated limb	2 patients reported persistent anterior knee pain 2 female patients were found to have knee-joint stiffness 3 male patients experienced a new episode of patellar dislocation
Schottle et al. [30]	Autograft: 15	Mean Kujala score improved from 53.3 (range, 31–76) to 85.7 (range, 55–100) at latest follow-up. 86% of patients had excellent or good results and 13% of patients had fair results. Previous surgery or mild trochlear dysplasia had no influence on the clinical outcomes. MPFL reconstruction reduces patellar tilt and may correct patellar alta	3 knees with persistent patellar apprehension
Torisuka et al. [31]	Autograft: 20	The average postoperative Kujala score was 96 ± 5 (84–100). According to Crosby-Insall criteria, all patients were graded as having excellent or good outcomes	No redislocation or patellar fracture 1 patient with patella infera due to arthrofibrosis
Vavalle et al. [32]	Autograft: 16	Both Kujala score and Lysholm scores were improved from 35.8 and 43.3 to 88.8 and 89.3, respectively	No recurrent episodes of dislocation or subluxation No complication occurred.
Wang et al. [33]	Autograft: 70 SB: 26 DB: 44	Both SB and DB MPFL reconstruction can effectively restore patellar stability and improve knee function. DB MPFL reconstruction showed better clinical outcomes compared to those of SB MPFL reconstruction. Patellar instability rates: SB: 19.2% and 26.9% at 12 months and 48 months (significant), DB: 2.27% and 4.54% at 12 months and 48 months, respectively (n. s.). Kujala score: SB: 87.8 ± 4.0 and 80.5 ± 3.6 at 12 months and 48 months (significant), DB: 92.3 ± 4.3 and 92.9 ± 2.5 at 12 months and 48 months, respectively (n. s.). Subjective questionnaire score SB: excellent and good rates were 88.5% and 80.8% at 12 months and at 48 months, DB: excellent and good rates were 97.7% and 95.5% at 12 months and at 48 months, respectively	Superficial wound infection occurred in 1 patient of each group. There was no deep vein thrombosis or ROM limitation in either group Patellar redislocation: 3 in SB group, none in DB group
Witonski et al. [34]	Autograft: 10	There were significant improvements found between preoperative and postoperative results in terms of clinical scales such as the Kujala score, the KOOS questionnaire, and most aspects of the SF-36 questionnaire	No recurrent dislocation
Zhao et al. [35]	Autograft: 45	When the MPFL reconstruction technique was compared to medial retinaculum plication, there were significantly favorable outcomes in IKDC, Lysholm and Kujala scores at the 60 months’ follow-up. IKDC subjective score improved from 46.3 ± 4.4 to 79.4 ± 6.8 at 60 months’ follow-up. Lysholm score improved from 52.1 ± 8.4 to86.9 ± 6.1 at 60 months’ follow-up. Kujala score improved from 68.9 ± 6.8 to 87.4 ± 5.7 at 60 months’ follow-up. Tegner score improved from 3.1 ± 1.9 to 5.7 ± 1.7 at 60 months’ follow-up	1 patient experienced an episode of redislocation. 3 patients experienced multiple episodes of patellar instability The failure rate of the MPFL reconstruction group was revealed as 8.9%

*SF-36* short form-36, *MPFL* medial patellofemoral ligament, *ROM* range of motion, *KOOS* Knee Injury and Osteoarthritis Outcome Score, *VR-12* Veterans RAND 12-Item Health Survey, *SB* single bundle, *DB* double bundle, *IKDC* International Knee Documentation Committee

### Assessment of methodological quality

Two investigators independently assessed the methodological quality of each study using the Coleman methodology score [36]. Each study was assessed using 10 methodological criteria, resulting in a final score ranging from 0 to 100. A perfect score of 100 indicated a study design that avoided the influence of chance, various biases, and confounding factors. Each author scored the methodological quality of each study twice, with a 10-day interval between assessments. Any disagreement between authors was resolved through discussion or review by a third investigator.

## Results

### Study identification

**Fig. 1:**
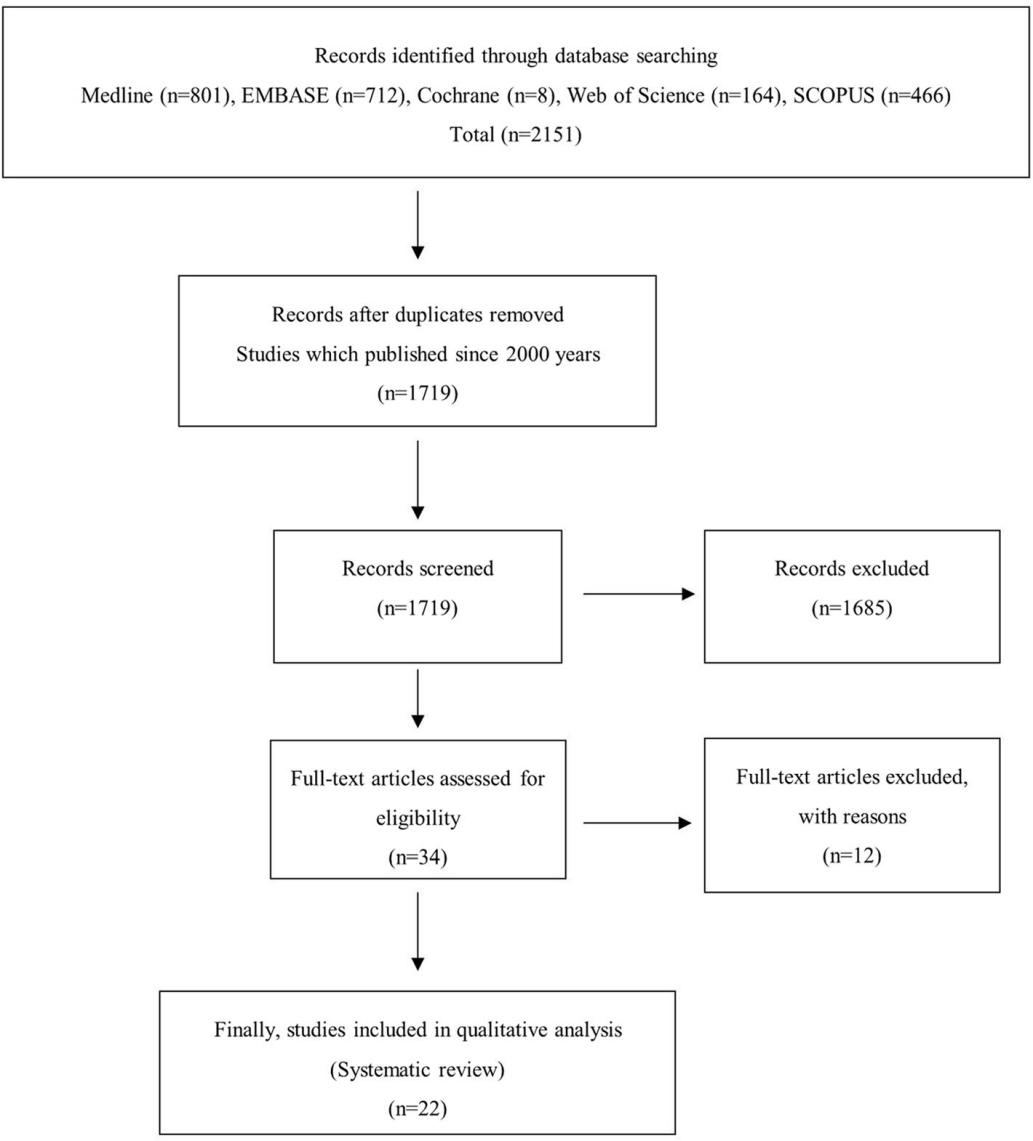
Flow diagram of the Preferred Reporting Items of Systematic Reviews and Meta-Analyses (PRISMA)

A total of 2151 relevant articles were initially identified. Of these, 432 were duplicates or published before the year 2000 in these databases. After screening the remaining 1719 articles using titles and abstracts, all but 34 were excluded because they were not relevant to the purpose of the present study. A full-text review of these 34 articles resulted in the exclusion of 12 articles because they did not meet the inclusion criteria. The remaining 22 clinical studies were included for data extraction and systematic review (Fig. 1) [9, 11–17, 22–32].

### Quality of included studies

The mean modified Coleman methodology score of these included studies was 78.1 ± 8.2 (range, 66 to 100). The results of the mean Coleman methodology score for each criterion are shown in Table 4.

**Table 4:** Overall Coleman methodology score for each criterion

Criteria (maximum score)	Mean	Standard deviation	Range
Part A			
1.Study size (10)	4.1	3.7	0–10
2. Mean follow-up (5)	5.0	0	5
3. Number of procedures (10)	10.0	0	10
4. Type of study (15)	3.9	6.5	0–15
5. Diagnostic certainty (5)	5.0	0	5
6. Surgery description (5)	5.0	0	5
7. Rehabilitation description (10)	9.6	2.1	0–10
Part B			
1.Outcome criteria (10)	10.0	0	10
2. Procedure for outcomes (15)	11.7	1.6	11–15
3. Selection process (15)	13.9	2.1	10–15
Coleman methodology score (100)	78.1	8.2	66–100

### Data abstraction (qualitative analysis)

#### Medial patellofemoral ligament reconstruction using an autograft

##### Kujala scores

Among 21 studies on MPFL reconstruction with autograft, 20 studies [9, 12–17, 22, 24–35] evaluated the Kujala score as a primary clinical outcome. Five randomized controlled trials (RCTs) [12, 24–26, 35] and 15 retrospective studies [9, 13–16, 22, 27–34] reported the Kujala score in MPFL reconstruction with an autograft, consisting of a total of 698 subjects. The reported range of postoperative mean Kujala score was from 80.5 to 96.0 points. There were significant differences between preoperative and postoperative Kujala scores in all 20 studies. Regarding surgical techniques, Wang et al. [33] found that double-bundle (DB) MPFL reconstruction showed better outcomes compared to single-bundle (SB) MPFL reconstruction. Kang et al. [24] reported that a Y-shape graft technique had favorable outcomes compared to a C-shape graft technique. Conversely, Niu et al. [26] and Zhao et al. [35] reported that MPFL reconstruction had significantly favorable Kujala scores compared to medial soft-tissue realignment surgery. However, Astur et al. [15] reported that there were no statistically significant differences in Kujala score between the endobutton and anchor fixation groups. Han et al. [16] reported that the results of the Kujala score were not associated with the presence of cartilage lesion, or sex.

##### Patellar instability (redislocation or subluxation)

Of 21 studies (714 subjects) on MPFL reconstruction with an autograft, only three studies [29, 33, 35] reported patellar redislocation after surgery. Redislocation occurred in 10 (1.4%) patients. Wang et al. [33] reported that patellar redislocation occurred more frequently in SB MPFL reconstruction compared to that in DB MPFL reconstruction. Although patellar redislocation did not occur, six studies [11, 13, 17, 22, 25, 30] reported that the persistent apprehension sign remained in their patients (10 patients, 1.4%).

##### Subjective results

Various clinical evaluation tools were used to investigate the subjective results after MPFL reconstruction using an autograft. For patients who underwent surgery, the percentage of good or excellent satisfaction ranged from 71.4 to 100.0% [9, 11, 12, 24, 25, 27, 28, 30, 31, 33]. Ma et al. [25] found that there were no significant differences in subjective questionnaire scores between medial retinaculum plasty and MPFL reconstruction with autograft groups. In terms of graft type, Kang et al. [24] reported a good or excellent rate of 97.5% in the Y-shape graft group compared to 83.3% in the C-shape graft group with significant difference.

##### Perioperative complications

Among 12 studies [9, 11, 15–17, 25–27, 29, 31–33] that dealt with perioperative complications, three [26, 27, 32] reported no perioperative complications after MPFL reconstruction with an autograft. Furthermore, six studies [9, 15, 16, 25, 29, 31] reported postoperative arthrofibrosis or limitations in the ROM. Flexion deficit was particularly prominent after the surgery. However, extension deficit was not found. Mikashima et al. [17] reported that there were two cases of patellar fracture in patients using an autograft. There were no infections or vascular problems such as deep vein thrombosis. However, one study [33] reported two cases of superficial wound infection.

#### Medial patellofemoral ligament reconstruction using allograft

##### clinical evaluation scales

MPFL reconstruction using allografts was also subjected to qualitative analysis. To evaluate clinical outcomes after MPFL reconstruction using allografts, only one study [23] was included. Using clinical knee evaluation scales, such as the KOOS (Knee Injury and Osteoarthritis Outcome Score), Lysholm, Tegner, and VR-12 (Veterans RAND 12-Item Health Survey), Dragoo et al. [23] have investigated whether MPFL repair is superior to MPFL reconstruction using a semitendinosus allograft. They found that there were no statistically significant differences in clinical outcomes between the two techniques. Thus, they concluded that MPFL repair or reconstruction with an allograft might lead to clinically acceptable results at 2-year follow-up.

##### Perioperative complications

One study reported perioperative complications after MPFL reconstruction with an allograft. Dragoo et al. [23] reported that, despite one report of postoperative recurrent dislocation in their MPFL repair cohort with a recurrence rate of 4%, there were no recurrent dislocations in any patients initially treated with MPFL reconstruction. Furthermore, there were no other surgical complications, including stiffness, infections, painful metalwork, or wound problems at the final follow-up.

## Discussion

In the present study, we assessed evidence from clinical studies that evaluated treatment outcomes after MPFL reconstruction using autograft or allograft materials. Although direct comparative studies were unavailable, the Kujala score and subjective results from the majority of studies indicated that an autograft for MPFL reconstruction yielded satisfactory clinical outcomes after MPFL reconstruction. However, no new outcome has been drawn from the use of allografts. The present study showed low rates of occurrence of perioperative complications in both groups. Furthermore, the rate of postoperative patellar instability was low at about 2.8%, and this value is similar to the pooled estimated value of postoperative redislocation rate observed in a previous review [37]. The results of the present systematic review partly supported our hypothesis that either autograft or allograft materials would yield favorable results for MPFL reconstruction. However, due to insufficient data description, direct comparison between both groups was not performed; thus, which technique yields better improvements in clinical outcome for MPFL reconstruction remains inconclusive.

Although many studies have investigated graft materials after anterior cruciate ligament (ACL) or posterior cruciate ligament (PCL) reconstruction, direct comparisons of clinical outcomes after MPFL reconstruction with autograft versus allograft are rarely reported. Only one study performed a direct comparison of an autograft versus an allograft for MPFL reconstruction [38]. However, that study was not included in the present review because it did not satisfy our inclusion due to the short-term follow-up period. In that study, Calvo Rodriguez et al. [38] reported that one patient received revision surgery due to poor positioning of the anchors. Furthermore, one patient had a non-displaced patellar fracture related to the bone tunnel and another patient had a flexion deficit. These three patients had received an allograft for MPFL reconstruction. Although three cases of perioperative complications occurred in their subjects, recurrent dislocations or graft-related complications were not observed. Ultimately, there were no significant differences in clinical outcomes between the two groups. Unlike that study, the present study did not conduct a direct comparison for MPFL reconstruction using autograft versus allograft. However, according to Sillanpaa et al.’s classification [39], almost all studies reporting the Kujala score were classified in the “good” category (85–94 points) for both groups. The results of the present study are similar to those of Calvo Rodriguez et al. Both studies revealed that MPFL reconstruction using both grafts had a favorable clinical outcome. To strengthen the evidence of these results, prospective (high-quality large-scale) comparative studies with similar clinical conditions are encouraged.

There is critical debate regarding the various surgical procedures concomitantly performed with MPFL reconstruction considering numerous predisposing factors, such as trochlear dysplasia, patellar height, graft types, rotational abnormalities of the tibia and femur, and the anterior tibial tuberosity to trochlear groove (TT-TG) distance [40]. To evaluate one independent factor, removing all confounding factors is ideal to reduce the risk of bias. For this reason, some authors have intentionally removed these confounding variables from consideration by narrowing their inclusion criteria [40]. However, strict control of all confounding factors affecting clinical outcomes is limited in practice. This concept is associated with “effectiveness” (heterogenous, more practical, “real-world”) studies in normal clinical conditions likely encountered in a real clinical trial [41]. Hence, the findings of the present study should be interpreted with great caution because the data involved were extracted from somewhat heterogenous studies. Besides, concomitant surgeries, such as lateral retinacular release and tibial tuberosity transfer, might increase surgery-related complications. Similarly, Buckens et al. [42] have considered that the heterogeneity of their series, with different concomitant procedures, might underestimate the real success of MPFL reconstruction. As such, our results imply that isolating MPFL reconstruction using autografts or allografts might produce more satisfactory results. If the authors want to focus on the “efficacy” (homogenous subjects, interventions, comparators, and outcome measures), future investigations should aim to establish more uniform criteria for selecting patients to undergo this procedure.

Based on the Coleman scales to assess the methodological quality, almost all the criteria in each study revealed a higher score. However, major sections of methodological deficiencies remained, including study size and type of the study. Theoretically, large-scale prospective studies would provide the rigorous control of potentially confounding factors. Thus, the present study critically appraised and synthesized the available evidence on this topic to provide a conclusion to a debatable issue. Further prospective studies are needed in the future to address methodological limitations. Screening and data extraction of the present study were carried out by two independent reviewers. This is one strength of our study. Although several recent systematic reviews have focused on ACL or PCL reconstruction with either an autograft or an allograft, less is known regarding autograft versus allograft for MPFL reconstruction. This study provides valuable evidence in support of MPFL reconstruction using an autograft or an allograft.

Despite its strengths, our study has some limitations. First, a relatively small number of prospective studies were included on each topic in our systematic review. There are few previously published original prospective studies with low risk of bias on this topic which is an absolute limitation. A review that is based on low-quality studies can affect conclusions. Second, in addition to demographic factors such as sex, age, and weight, technical factors regarding surgical methods also need to be controlled, including the transpatellar tunnel technique or non-transpatellar tunnel technique, various graft types, and fixation methods because they might affect the results following MPFL reconstruction. Third, we did not fully consider concomitant procedures that could affected clinical outcomes, such as tibial tuberosity transfer, lateral retinacular lengthening, or trochleoplasty. In other words, the methodologies of the studies included here are different from each other; they have heterogeneity. Due to such heterogeneity and the absence of direct comparative studies, we could not compare these two graft materials using statistical methods or conclude which graft material was better.

## Conclusions

Although many studies showed favorable clinical results for MPFL reconstruction using an autograft, the clinical results of MPFL reconstruction using an allograft have not yet been sufficient to achieve a meaningful clinical result due to low evidence. Direct comparisons were not conducted because there were very few studies on allografts; thus, further research in this area should be performed in the future.

## Additional file

Additional file 1: Electronic search 373 strategy for each database. (DOCX 19 kb)

## Data Availability

Not applicable.

## Abbreviations

ACL: Anterior cruciate ligament
DB: Double bundle
KOOS: Knee Injury and Osteoarthritis Outcome Score
MPFL: Medial patellofemoral ligament
PCL: Posterior cruciate ligament
RCT: Randomized controlled trial
ROM: Range of motion
SB: Single bundle
SD: Standard deviation
TT-TG: Tibial tuberosity-trochlear groove
VR-12: Veterans RAND 12-Item Health Survey
