# Supplementary material for: Medial patellofemoral ligament reconstruction using an autograft or allograft for patellar dislocation: a systematic review
Source: Knee Surg Relat Res. 2019 Aug 23;31:8. doi: 10.1186/s43019-019-0008-0 (PMC7219573; doi:10.1186/s43019-019-0008-0)
Supplement: Supplementary file 1 — Electronic search 373 strategy for each database. (DOCX 19 kb) [file 43019_2019_8_MOESM1_ESM.docx]

**Additional file 1**

MEDLINE

1. “Medial patellofemoral ligament”[tiab] OR “MPFL”[tiab], n=628
2. “Recurrent patellar dislocation” [tiab] OR “recurrent patellar instability”[tiab], n=280
3. "Patellar dislocation"[Mesh], n=795
4. 1 OR 2 OR 3, n=1248
5. Medial reefing[tiab] OR Medial augmentation[tiab] OR Medial plication[tiab] OR Medial imbrication[tiab] OR Quadricepsplasty[tiab] OR Proximal realignment[tiab], n=844
6. Single-Bundle[tiab] OR Double-Bundle[tiab] OR ((Single[tiab] OR Double[tiab]) AND Bundle[tiab]), n=4974
7. 5 OR 6, n=5809
8. 4 AND 7, n=866
9. 9 NOT "review"[Publication Type] OR "review literature as topic"[MeSH Terms], n=801

EMBASE

1. 'knee ligament'/de OR 'knee cruciate ligament'/exp, n=14755
2. "Medial patellofemoral ligament":ab,ti, n=636
3. “Patellar dislocation”:ab,ti, n=872
4. 1 OR 2 OR 3, n=15906
5. Medial reefing:ab,ti OR Medial augmentation:ab,ti OR Medial plication:ab,ti OR Medial imbrication:ab,ti OR Quadricepsplasty:ab,ti OR Proximal realignment:ab,ti, n=322
6. Single-Bundle:ab,ti OR Double-Bundle:ab,ti OR ((Single:ab,ti OR Double:ab,ti) AND Bundle:ab,ti), n=6059
7. 5 OR 6, n=6381
8. 4 AND 7, n=772
9. 8 NOT ('conference review'/it OR 'review'/it), n=712

COCHRANE

1. “Medial patellofemoral ligament” OR “MPFL”:ti,ab,kw, n=32
2. MeSH descriptor: [Patellar dislocation] explode all trees, n=35
3. 1 OR 2, n=57
4. Medial reefing OR Medial augmentation OR Medial plication OR Medial imbrication OR Quadricepsplasty OR Proximal realignment:ti,ab,kw, n=43
5. Single-Bundle OR Double-Bundle OR ((Single OR Double) AND Bundle):ti,ab,kw, n=445
6. 4 OR 5, n=485
7. 3 AND 6, n=10
8. 7/trials, n=8

Web Of Science (WOS)

1. TOPIC: (“Medial patellofemoral ligament” OR “MFPL” OR “Patellar dislocation”) OR TITLE: (“Medial patellofemoral ligament” OR “MFPL” OR “Patellar dislocation”), n=1716
2. TOPIC: (Medial reefing OR Medial augmentation OR Medial plication OR Medial imbrication OR Quadricepsplasty OR Proximal realignment OR Single-Bundle OR Double-Bundle OR ((Single OR Double) AND Bundle)) OR TITLE: (Medial reefing OR Medial augmentation OR Medial plication OR Medial imbrication OR Quadricepsplasty OR Proximal realignment OR Single-Bundle OR Double-Bundle OR ((Single OR Double) AND Bundle)), n=15098
3. 1 AND 2, n=175
4. 3 Refined by: [excluding] DOCUMENT TYPES: ( REVIEW ), n=164

SCOPUS

1. TITLE-ABS ( "Medial patellofemoral ligament" OR "MFPL" ), n=701
2. INDEXTERMS (“ medial patellofemoral ligament” ), n=169
3. 1 OR 2, n=725
4. TITLE-ABS ("Patellar dislocation" ), n=1051
5. INDEXTERMS ( “patellar dislocation” ), n=743
6. “Medial reefing” OR “Medial augmentation” OR “Medial plication” OR “Medial imbrication” OR “Quadricepsplasty” OR “Proximal realignment” OR “Single-Bundle” OR “Double-Bundle” OR ((“Single” OR “Double”) AND “Bundle”), n=112414
7. 4 OR 5 OR 6, n=113608
8. 3 AND 7, n=466
9. 8 AND EXCLUDE ( DOCTYPE , "re" ), n=466
